# Advances and challenges of mesenchymal stem cells for pregnancy-related diseases

**DOI:** 10.1038/s41423-021-00707-7

**Published:** 2021-06-25

**Authors:** Yan-Hong Li, Di Zhang, Mei-Rong Du

**Affiliations:** 1 NHC Key Lab of Reproduction Regulation (Shanghai Institute of Planned Parenthood Research), Hospital of Obstetrics and Gynecology, Fudan University Shanghai Medical College, Shanghai, China; 2 Shanghai Key Laboratory of Female Reproductive Endocrine Related Diseases, Shanghai, China; 3 Department of Obstetrics and Gynecology, Shanghai Ninth People’s Hospital, Shanghai Jiao Tong University School of Medicine, Shanghai, China; 4 Department of Obstetrics and Gynecology, Guangzhou First People’s Hospital, School of Medicine, South China University of Technology, Guangzhou, China; 5 State Key Laboratory of Quality Research in Chinese Medicine and School of Pharmacy, Macau University of Science and Technology, Macau SAR, China

**Keywords:** Immunological disorders, Immunotherapy

Mesenchymal stem cells (MSCs) with pluripotency, wide origin and strong migration ability, but low immunogenicity and lack of ethical controversies, have been intensely investigated for clinical applications within the last decades. Our previously published data in this issue of Cellular and Molecular Immunology demonstrated that adoptive transfer of MSCs can prevent fetal loss in lipopolysaccharide-induced and spontaneous abortion models via a paracrine effect and a cell contact-dependent manner. MSC-derived TSG-6 promotes the transformation of pro-inflammatory macrophages (M1) to anti-inflammatory ones (M2), as well as inhibits the proliferation and inflammatory response of CD4^+^ T cells. MSC-M1 contact increases the production of TSG-6 and thus enhances TSG-6-mediated paracrine effect. In addition, CD200 on MSCs interacting with CD200R on M1 played an indispensable role in the transition of M1 to M2 by MSCs. Notably, M1 exerted a promotion effect on the CD200 expression of MSCs. Therefore, soluble factor, TSG-6 cooperates with the MSC-M1 contact, involved in the immunosuppression by MSCs against abortion.^1^

The role of MSCs in the treatment of miscarriage or recurrent pregnancy loss is confirmed by emerging evidence (Fig. 1). The current research proves that in addition to macrophages and CD4^+^ T cells, other immune cells at the maternal–fetal interface including NK, DC cells, and Treg are also involved in the underlying mechanisms. Fatemeh et al. showed that adipose-derived (AD) MSCs prevented abortion by inhibiting the excessive infiltration of peripheral NK cells to the decidua along with upregulating IL4 and IL10 production of decidual NK cells and downregulating IFN-γ production.^2^ As regard to decidual DCs, recovered frequency as well as a relative immature phenotype with tolerogenic function was induced in abortion-prone mouse models after AD-MSCs administration.^3^ Besides, MSC therapy protected fetus in abortion-prone mice through promoting Treg expansion in inguinal lymph nodes and expression of Treg-related genes in decidua and placenta.^4^ In addition to newly revealed mechanisms, new cell sources and application methods have also been reported in MSC therapy for abortion. MSCs used in abortion therapy are isolated from human umbilical cord (huc) and Wharton jelly or mouse-derived bone marrow (BM) and adipose tissue. Precursors of human decidual stromal cells (DSCs) are the newly defined decidual MSCs and possess immunoregulatory activities to treat an immune-based mouse model of recurrent spontaneous abortion.^5^ Besides, BMSCs-derived exosomes injection instead of MSCs administration is proved to be an effective strategy to modulate the function of decidual T cells and macrophages, thereby ameliorating pregnancy outcomes in abortion-prone mice.^6^

**Fig. 1:**
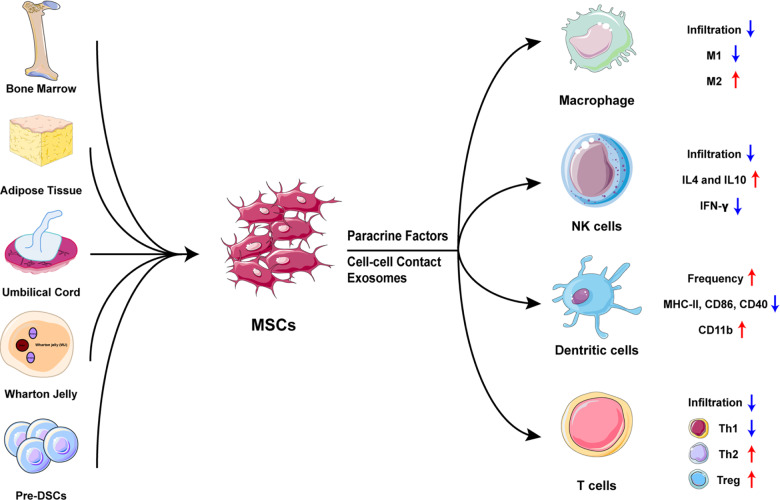
Mechanisms of MSC-based therapy for pregnant loss. MSCs isolated from different tissues, including BM, AD, UC, WJ, and the precursors of decidual stromal cells, are administrated to abortion animal models and exert multifaced immunomodulation on maternal immune cells via paracrine factors, cell–cell contact and exosomes, suppressing excessive activated immune responses and intensified inflammation associated with pregnant loss

Except for RPL, preeclampsia is another intensively investigated pregnancy-related disease about its treatment based on MSC therapy. PE is a complication characterized by high blood pressure, proteinuria, and edema, occurring after 20 weeks of pregnancy. MSC therapy opens opportunities for the development of novel treatment of PE. In various murine and rat PE models, MSC transplantation demonstrates promising therapeutic effects.^7–9^ By modulating the balance of anti-inflammatory and pro-inflammatory cytokines in decidua and placenta, the biological behavior of trophoblasts (like proliferation, apoptosis, invasion, and migration), and placental vascular remodeling and angiogenesis, MSCs are potent to improve placental function and relieve PE symptoms. More recently, extracellular vesicles (EVs) isolated from MSCs are used instead and have similar therapeutic effects in PE as MSCs themselves, providing a novel insight for PE treatment (Table S1). For instance, EV-derived microRNA-18b was reported to ameliorate PE by enhancing trophoblasts proliferation and migration. MSCs-derived exosomal microRNA-18b-3p targeted LEP and thus inhibited placental inflammatory cytokines expression and cell apoptosis to prevent PE. Collectively, a promising therapeutic value of MSCs in PE is suggested by these preclinical researches.

While MSCs-based therapy in other diseases such as cancers, graft versus host disease, autoimmune disorders, has been extensively studied and even achieved exciting results in late-stage clinical trials, there are only a small number of studies performed in RPL and PE models. Much more efforts should be devoted for the translation of MSCs to the clinical stage to help women with pregnancy-related diseases. MSC-EVs are now widely accepted as alternatives to MSCs for new cell-free therapeutic strategies in various clinical trials. Cell-free therapy based on MSC-EVs avoids emboli formation, undesired differentiation, infection transmission, potential tumorigenicity, and ethical obstacles. Moreover, MSC-EVs are more stable for storage and transport, and easier for the quality and dosage control, thus it is safer and more convenient to apply them into clinical conditions.^10,11^ However, the role of MSC-EVs in RPL and PE and the specific mechanisms are just explored by few in vitro experiments and animal models, far less thoroughly investigated. Thus, a series of questions need to be addressed to help MSC-EVs advance forward clinical applications in pregnancy-related diseases. For example, no study compares EVs from different MSC sources, such as BM-MSCs, hucMSCs, AD-MSCs, placental-MSCs, in terms of their efficacy, molecular mechanisms, or possible therapeutic risks in pregnancy-related diseases. Therefore, the first question for MSC-EVs application in pregnancy-related diseases is to identify the best MSC source. Whether MSC-EVs can be combined with carriers such as scaffold materials in order to extend their activity in the uterus? Use 3D culture and other novel methods to increase the production of MSC-EVs, and manufacture individualized MSC-EVs for RPL or PE according to molecular mechanism of pathology? These are the possible points of future research and may pave the way to MSC-EVs clinical translation in pregnancy-related diseases. Besides the rapid development of EV-based MSC therapy, genetic modification of MSCs to obtain over-expressed antitumor genes or therapeutic factors has gradually became a new approach of MSC utility.^12,13^ To date, no relevant research has been reported in pregnancy-related diseases. Whether gene editing can be considered to promote the homing of MSCs to uterus, reinforce the anti-inflammatory immunomodulation, or enhance the pro-angiogenesis function, so as to provide better therapeutic effect of MSCs for pregnancy-related diseases? One may anticipate that by figuring out the mechanisms and taking use of the latest culture methods, manipulative strategies, and application modes, the development of MSCs-based therapies for pregnancy-related diseases may experience more rapid advancement.

## Supplementary information

Supplemental Table 1
