# Supplementary material for: Advances and challenges of mesenchymal stem cells for pregnancy-related diseases
Source: Cell Mol Immunol. 2021 Jun 25;18(8):2075–7. doi: 10.1038/s41423-021-00707-7 (PMC8322408; doi:10.1038/s41423-021-00707-7)
Supplement: Supplementary file 1 — Supplemental Table 1 [file 41423_2021_707_MOESM1_ESM.ppt]

## Slide 1
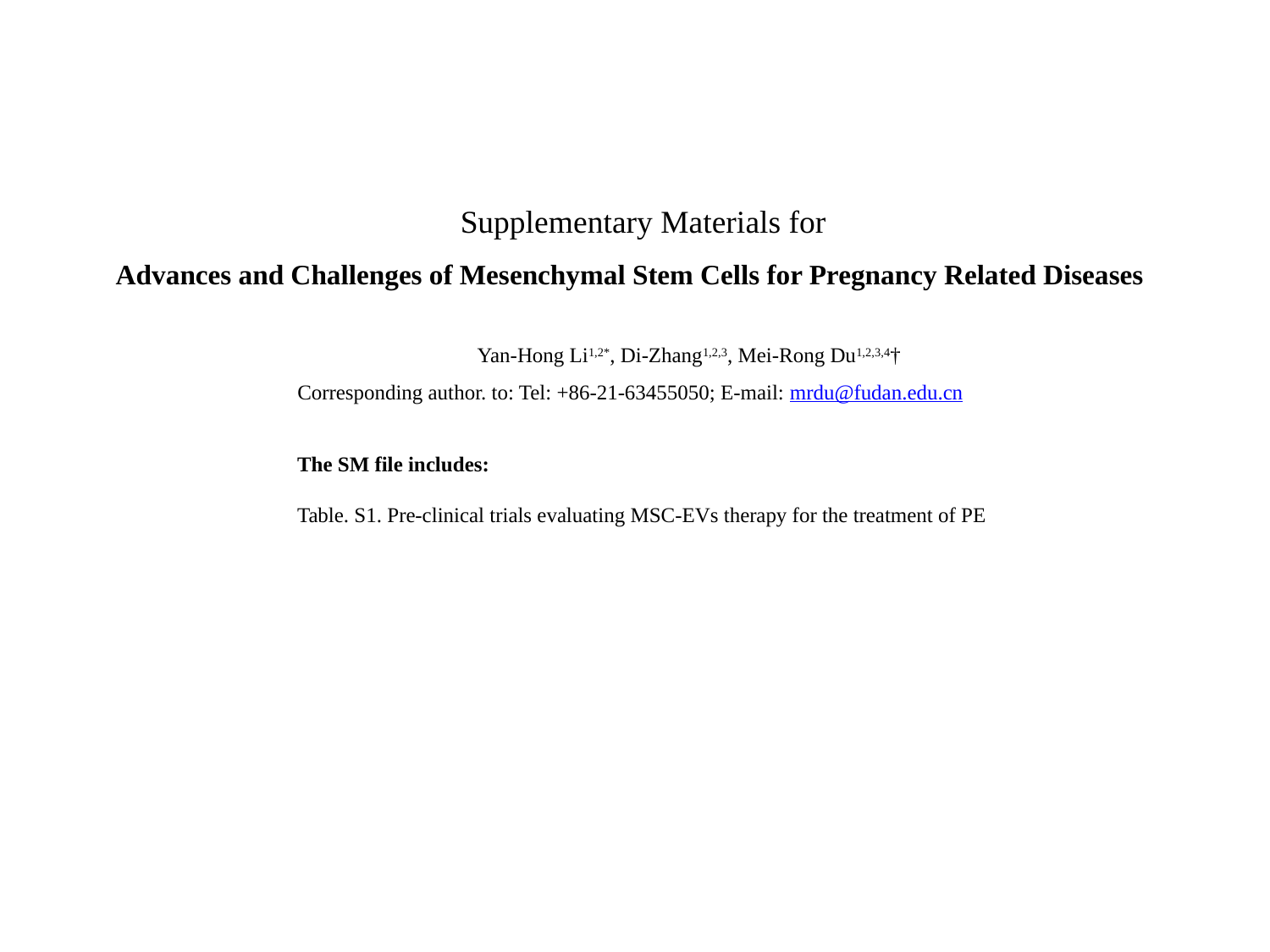

Supplementary Materials for
Advances and Challenges of Mesenchymal Stem Cells for Pregnancy Related Diseases
Yan-Hong Li1,2*, Di-Zhang1,2,3, Mei-Rong Du1,2,3,4†
Corresponding author. to: Tel: +86-21-63455050; E-mail: mrdu@fudan.edu.cn
The SM file includes:
Table. S1. Pre-clinical trials evaluating MSC-EVs therapy for the treatment of PE

## Slide 2
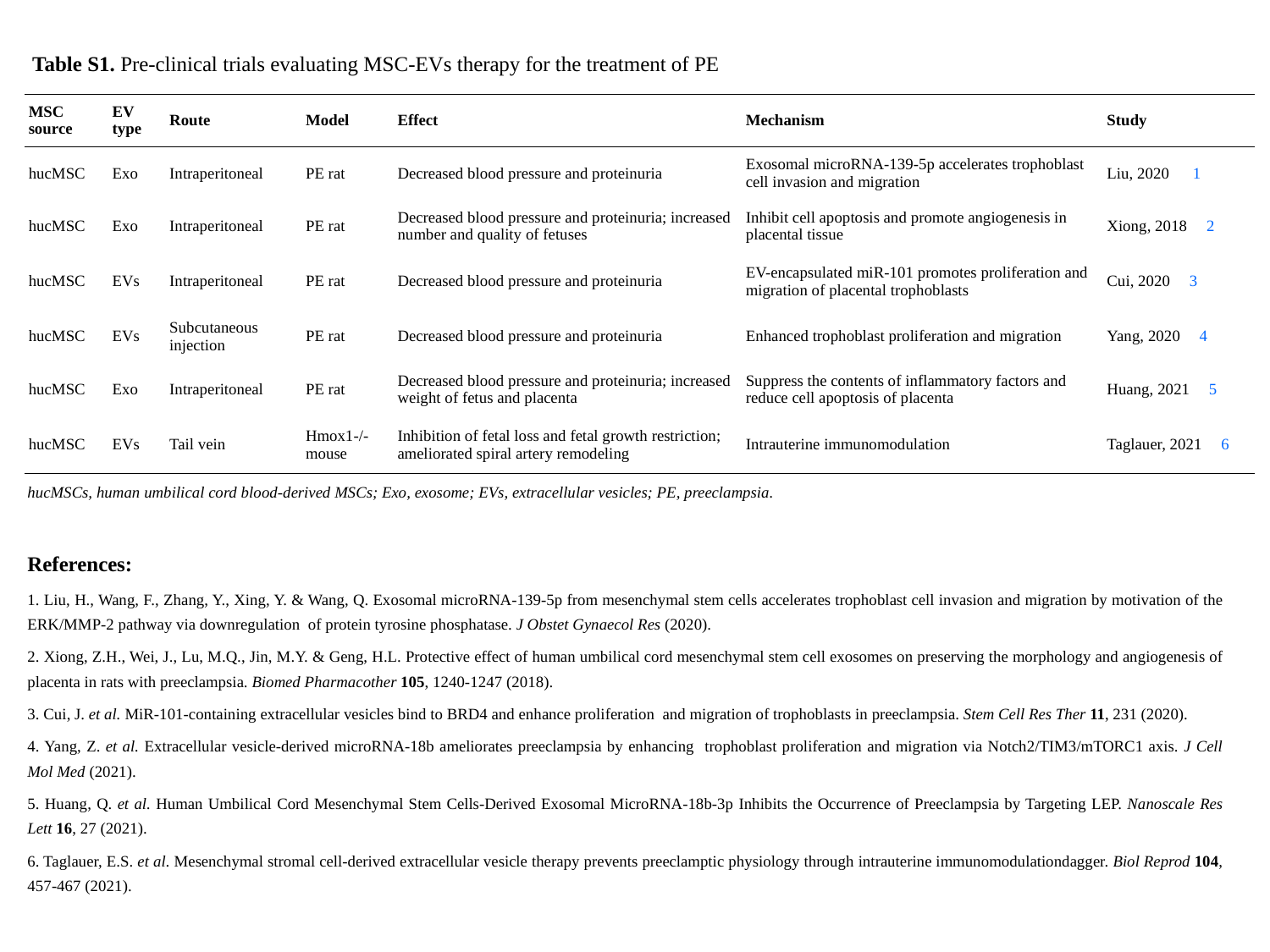

Table S1. Pre-clinical trials evaluating MSC-EVs therapy for the treatment of PE
| MSC source | EV type | Route | Model | Effect | Mechanism | Study |
| --- | --- | --- | --- | --- | --- | --- |
| hucMSC | Exo | Intraperitoneal | PE rat | Decreased blood pressure and proteinuria | Exosomal microRNA-139-5p accelerates trophoblast cell invasion and migration | Liu, 2020 ［1］ |
| hucMSC | Exo | Intraperitoneal | PE rat | Decreased blood pressure and proteinuria; increased number and quality of fetuses | Inhibit cell apoptosis and promote angiogenesis in placental tissue | Xiong, 2018［2］ |
| hucMSC | EVs | Intraperitoneal | PE rat | Decreased blood pressure and proteinuria | EV-encapsulated miR-101 promotes proliferation and migration of placental trophoblasts | Cui, 2020［3］ |
| hucMSC | EVs | Subcutaneous injection | PE rat | Decreased blood pressure and proteinuria | Enhanced trophoblast proliferation and migration | Yang, 2020［4］ |
| hucMSC | Exo | Intraperitoneal | PE rat | Decreased blood pressure and proteinuria; increased weight of fetus and placenta | Suppress the contents of inflammatory factors and reduce cell apoptosis of placenta | Huang, 2021［5］ |
| hucMSC | EVs | Tail vein | Hmox1-/- mouse | Inhibition of fetal loss and fetal growth restriction; ameliorated spiral artery remodeling | Intrauterine immunomodulation | Taglauer, 2021［6］ |
hucMSCs, human umbilical cord blood-derived MSCs; Exo, exosome; EVs, extracellular vesicles; PE, preeclampsia.
References:
1. Liu, H., Wang, F., Zhang, Y., Xing, Y. & Wang, Q. Exosomal microRNA-139-5p from mesenchymal stem cells accelerates trophoblast cell invasion and migration by motivation of the ERK/MMP-2 pathway via downregulation of protein tyrosine phosphatase. J Obstet Gynaecol Res (2020).
2. Xiong, Z.H., Wei, J., Lu, M.Q., Jin, M.Y. & Geng, H.L. Protective effect of human umbilical cord mesenchymal stem cell exosomes on preserving the morphology and angiogenesis of placenta in rats with preeclampsia. Biomed Pharmacother 105, 1240-1247 (2018).
3. Cui, J. et al. MiR-101-containing extracellular vesicles bind to BRD4 and enhance proliferation and migration of trophoblasts in preeclampsia. Stem Cell Res Ther 11, 231 (2020).
4. Yang, Z. et al. Extracellular vesicle-derived microRNA-18b ameliorates preeclampsia by enhancing trophoblast proliferation and migration via Notch2/TIM3/mTORC1 axis. J Cell Mol Med (2021).
5. Huang, Q. et al. Human Umbilical Cord Mesenchymal Stem Cells-Derived Exosomal MicroRNA-18b-3p Inhibits the Occurrence of Preeclampsia by Targeting LEP. Nanoscale Res Lett 16, 27 (2021).
6. Taglauer, E.S. et al. Mesenchymal stromal cell-derived extracellular vesicle therapy prevents preeclamptic physiology through intrauterine immunomodulationdagger. Biol Reprod 104, 457-467 (2021).
